# Association of β-Blocker Use at Time of Radical Prostatectomy With Rate of Treatment for Prostate Cancer Recurrence

**DOI:** 10.1001/jamanetworkopen.2021.45230

**Published:** 2022-01-26

**Authors:** Shivanthe Sivanesan, Kristin Austlid Taskén, Helene Hartvedt Grytli

**Affiliations:** 1 Department of Urology, Oslo University Hospital, Oslo, Norway; 2 Institute of Cancer Research, Oslo University Hospital, Oslo, Norway; 3 Institute of Clinical Medicine, University of Oslo, Oslo, Norway

## Abstract

**Importance:**

The perioperative period has gained attention as a window of opportunity to prevent cancer recurrence. Evidence in support of a role for nonselective β-blockers (nsBBs) in cancer treatment is increasing, and counteracting cancer recurrence associated with perioperative stress and catecholamine is one of the suggested mechanisms of action.

**Objective:**

To explore whether use of nsBBs at the time of radical prostatectomy is associated with a lower rate of treatment for prostate cancer recurrence.

**Design, Setting, and Participants:**

This cohort study analyzed prospectively collected data from the Cancer Registry of Norway, Norwegian Patient Registry, Norwegian Prescription Database, and Norwegian Cause of Death Registry. Of 12 298 eligible patients, this study included 11 117 treatment-naive patients with prostate cancer (ie, no prior hormonal therapy, radiotherapy, or chemotherapy) who underwent radical prostatectomy in Norway from January 1, 2008, to December 31, 2015, with a minimum progression-free follow-up of 6 months. Data analysis was performed from April 20, 2020, to April 30, 2021.

**Exposures:**

Use of nsBBs and selective β-blockers (sBBs) at time of radical prostatectomy.

**Main Outcomes and Measures:**

Treatment for cancer recurrence after radical prostatectomy (defined as initiation of hormonal therapy, radiotherapy, or chemotherapy) or, if no treatment was identified, cancer-specific mortality.

**Results:**

The study included 11 117 men with prostate cancer (median [IQR] age at radical prostatectomy, 64.8 [60.4-68.3] years). Of these, 1622 (14.6%) later received treatment for cancer recurrence during a median follow-up of 4.3 years (IQR, 2.4-6.3 years). Use of nsBBs at time of surgery among 209 patients was significantly associated with a lower risk of treatment for cancer recurrence (adjusted hazard ratio [aHR], 0.64; 95% CI, 0.42-0.96; *P* = .03). No such association was observed for use of sBBs (aHR, 0.96; 95% CI, 0.84-1.11; *P* = .62). Subanalyses with (1) relaxed inclusion criteria allowing for inclusion also of patients with early progression (within 6 months) and (2) only the healthiest patients (Eastern Cooperative Oncology Group performance status of 0) supported the main findings.

**Conclusions and Relevance:**

In this cohort study, use of nsBB but not sBBs at the time of radical prostatectomy was associated with less treatment initiation for cancer recurrence. This finding, together with accumulated preclinical and clinical evidence, provides a foundation for initiation of an interventional study.

## Key Points

**Question:**

Is incidental use of a nonselective β-blocker (nsBB) at the time of surgery associated with a reduced rate of treatment for prostate cancer recurrence in patients who have undergone radical prostatectomy?

**Findings:**

In this cohort study of 11 117 patients with prostate cancer undergoing radical prostatectomy, use of an nsBB at the time of surgery was significantly associated with a lower rate of treatment for prostate cancer recurrence.

**Meaning:**

These results suggest that perioperative use of an nsBB at the time of radical prostatectomy may reduce the risk of prostate cancer recurrence, although randomized clinical trials are needed to answer this question.

## Teaser

This cohort study of men who have undergone radical prostatectomy assesses the association between incidental nonselective β-blocker use and treatment for prostate cancer recurrence.

## Introduction

Radical prostatectomy (RP) is a standard treatment for localized prostate cancer and is increasingly considered a treatment option also in locally advanced prostate cancer.^1,2^ Unfortunately, a significant proportion of patients experience cancer recurrence that requires further oncological treatment, most commonly radiotherapy and hormonal therapy, both of which put the patient at risk of treatment-related adverse effects.^3,4,5^

The perioperative period is receiving increased attention as a platform to inhibit cancer progression.^6,7,8^ Increased catecholamine levels attributable to surgical tissue trauma, anesthetic agents, and psychological stress^9,10^ may affect cancer cell plasticity, the surrounding vasculature, and the immune cell landscape, promoting metastasis and growth of residual disease.^6,7,8,10,11,12,13^ Prior studies^14,15^ have established the presence of disseminated tumor cells in patients with localized prostate cancer. Of interest, these cells may be activated from dormancy by catecholamines, consequently facilitating distant progression.^16^

β_2_-Adrenergic receptor (β_2_-AR) antagonists, including nonselective β-blockers (nsBBs) that block both β_1_- and β_2_-AR, have been found to inhibit these effects in several preclinical models.^13,17,18,19^ This inhibition has not been demonstrated to the same extent for selective BBs (sBBs), which target only β_1_-ARs.^18^ In a randomized clinical trial^20^ in patients undergoing mastectomy for breast cancer, perioperative treatment with the nsBB propranolol reduced biomarkers associated with metastasis. Currently, a phase 2 randomized clinical trial^21^ investigating the effect of perioperative nsBBs on apoptosis in prostate cancer is ongoing.

Associations between the use of BBs and oncological outcomes have been investigated in many epidemiological studies,^22,23,24^ including in prostate cancer specifically. Some have reported that BB use is associated with improved outcomes^22,23^; others have observed no association^24,25,26^ or even worse outcome.^27^ Inconsistencies may reflect variations in BB exposure definitions and end points, different availability of clinical variables, and inconsistency in differentiating between sBBs and nsBBs. In this study, we coupled data from national registries to identify a large cohort of men undergoing RP, allowing us to analyze nsBB and sBB use separately. We hypothesized that use of nsBBs at the time of surgery would be associated with less treatment for cancer recurrence after RP.

## Methods

This cohort study was approved by the Norwegian Regional Committee for Medical Health Service Ethics, the Norwegian Data Inspectorate, and each of the relevant registries. The national registries are regulated according to the Personal Health Data Registries Act. The collection of data by the registries used in this study does not require consent, whereas disclosure of information to the project was made possible through an exemption from the duty of confidentiality granted by the Norwegian Regional Committee for Medical Health Service Ethics. The personal information is personally identifiable within the registries and pseudonymized before disclosing for research purposes. This study follows the Strengthening the Reporting of Observational Studies in Epidemiology (STROBE) reporting guideline.^28^

### Data Sources

Using the unique Norwegian national identity number, assigned for life to everyone born or settled in Norway, we obtained and coupled data from the Cancer Registry of Norway, Norwegian Patient Registry, Norwegian Prescription Database, and Norwegian Cause of Death Registry to enable capture of patient and cancer characteristics for all men diagnosed with prostate cancer in Norway. Information provided by the individual registries is presented in Table 1.

**Table 1.** Overview of the Registries Used in This Study

National registry	Use	Additional information
Cancer Registry of Norway	Identify men diagnosed with prostate cancer in 2004-2015 and acquire patient and cancer characteristics, including age, ECOG performance status, time of diagnosis, TNM stage, ISUP grade, and PSA levels	Established in 1951 and contains clinical information on all patients diagnosed with prostate cancer in Norway; all physicians involved in cancer care are by law^29^ obliged to report cancer cases to the Cancer Registry of Norway
Norwegian Patient Registry	Identify men treated with RP and RT for prostate cancer and timing of this treatment and identify, if and when, HT and chemotherapy for prostate cancer were delivered to patients at national hospitals and affiliated specialist clinics (ie, not filled prescriptions)	Established in 2008 and provides data on patients treated at national hospitals and affiliated specialist clinics; diagnosis is labeled by *ICD-10* codes, and procedures (medical, surgical, and radiologic) are labeled in accordance with the national procedure coding system; reporting to the Norwegian Patient Registry, when providing health care, is mandatory by law^30^ and necessary for hospitals or specialist clinics to receive reimbursements
Norwegian Prescription Database	Identify time of exposure to BB subgroups, acetylsalicylic acid, metformin, and statins and identify, if and when, patients received HT, including LHRH agonists and antagonists, first- and second-generation antiandrogens, abiraterone, and docetaxel	Established in 2004 and contains detailed data on all filled prescriptions on pharmacy-dispensed drugs; reported according to the Anatomical Therapeutic Chemical classification system
Norwegian Cause of Death Registry	All-cause mortality and prostate cancer–specific mortality	Established in the 1960s and contains all causes of deaths in Norway; based on mandatory death certificate filed by physicians for all deaths that occur in Norway^31^

Abbreviations: BB, β-blocker; ECOG, Eastern Cooperative Oncology Group; HT, Hormonal therapy; *ICD-10, International Statistical Classification of Diseases and Related Health Problems, Tenth Revision*; ISUP, International Society of Urological Pathology; LHRH, luteinizing hormone–releasing hormone; PSA, prostate-specific antigen; RP, radical prostatectomy; RT, radiotherapy.

### Study Design, Setting, and Patients

All men diagnosed with prostate cancer in Norway from January 1, 2004, to December 31, 2015, were included in this study to enable identification of men who had undergone RP between January 1, 2008, and December 31, 2015 (end of follow-up). Data analysis was performed from April 20, 2020, to April 30, 2021. The exclusion criteria prior hormonal therapy, radiotherapy, or chemotherapy for prostate cancer and known metastasis before or at the time of RP left 12 298 eligible patients.

To exclude patients with suspected surgical failure and/or unrecognized metastatic disease at the time of RP, in the absence of information on postoperative prostate-specific antigen (PSA) levels, surgical margins, and any radiologic imaging, follow-up was started 6 months after RP. Consequently, patients receiving treatment for recurrence or unavailable for follow-up within the first 6 months were excluded (Figure).

**Figure.**
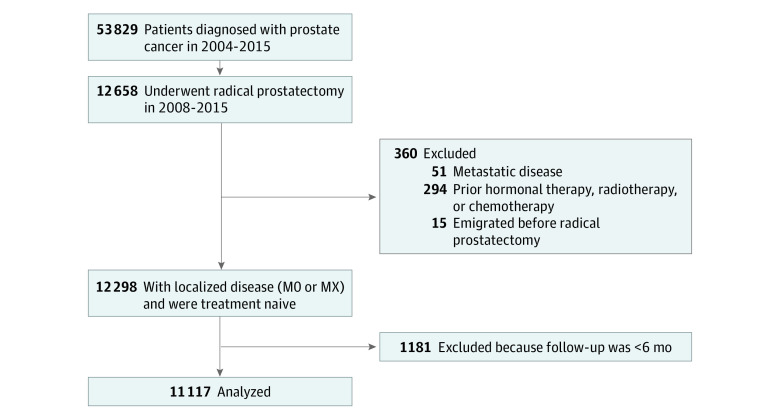
Participant Flow Diagram

### Variables, Exposures, and End Points

The Anatomical Therapeutic Chemical (ATC) classification system was used to identify all filled prescriptions of nsBBs (C07AA, -AG), sBBs (C07AB, -BB), acetylsalicylic acid (B01AC06, N02BA01, N02BA11), metformin (A10BA02), and statins (C10AA, -BA) from January 1, 2004, to December 31, 2015. Patients were considered users of nsBBs, sBBs, acetylsalicylic acid, metformin, and/or statins at time of surgery if they had filled a prescription within the past 100 days before RP (eFigure in the Supplement). Patients who had filled prescriptions for both nsBBs and sBBs (n = 21) were treated as nsBB users in the statistical analyses. Specific ATC codes, generic names, and number of users are provided in eTable 1 in the Supplement.

The end point, treatment of prostate cancer recurrence after RP, was used as a surrogate end point for recurrence. This end point was defined as initiation of hormonal therapy (ATC code L02), radiotherapy, chemotherapy for prostate cancer (whichever came first), or prostate cancer–specific death if no recurrence treatment was identified. Time of initiation of hormonal therapy was defined as the time of the first filling of a hormonal therapy prescription. For radiotherapy and chemotherapy, time of initiation was registered at the time of the first hospital admission.

### Statistical Analysis

The Mann-Whitney *U* test was used to compare continuous variables between groups, whereas the Pearson χ^2^ test and linear-by-linear association test for trends were used for categorical and ordinal variables, respectively. Continuous variables are presented as medians (IQRs) and categorical variables as numbers (percentages). The PSA values and clinical T stage were stratified before analysis, in accordance with the current European Association of Urology risk groups for biochemical recurrence.^32^ A 2-sided *P* < .05 was considered to indicate statistical significance.

Although missing data were assumed to be missing completely at random, they were addressed by performing multiple imputation, with fully conditional specification,^33^ and logistic and linear regression for categorical and scaled variables, respectively. Fifty separate imputed data sets were generated by multiple imputation; included variables and measures of missingness are detailed in eTable 2 in the Supplement.

The association between nsBB or sBB use at the time of RP and treatment for recurrence was analyzed by Cox proportional hazards regression modeling on both pooled data (ie, multiple imputed and complete cases pooled) and complete cases only. A competing risk analysis, treating death from causes other than prostate cancer as the competing risk, was performed to address potential bias caused by death that occurred before sufficient time to receive treatment for progression. Sensitivity analyses with relaxed exclusion criteria were performed with no exclusion for early treatment after RP and exclusion only of patients who received treatment within the first 3 months (90 days) after RP.

In addition, analyses stratified on duration of nsBB use before RP (<6 vs ≥6 months) and current use vs previous use only were performed to investigate associations with extended prior use (eFigure in the Supplement). Potential confounding from covariates not included in the final model (such as co-use of acetylsalicylic acid, metformin, and/or a statin) was assessed by analyzing them in the main Cox proportional hazards regression model, both with and without BB use included. Statistical analyses were performed with SPSS Statistics software, version 27.0 (IBM Inc) and Stata/MP software, version 16.1 (StataCorp LLC) (used for competing risk analysis only).

## Results

### Patient Characteristics

A total of 11 117 men (median [IQR] age at surgery, 64.8 [60.4-68.3] years) were included in the study (Figure). The registries used in this study contain no information on race or ethnicity. Treatment for recurrence was received by 1622 patients (14.6%) after a median follow-up after RP of 4.3 years (IQR, 2.4-6.3 years). A total of 209 (1.9%) patients were defined as nsBB users and 1511 (13.6%) as sBB users at the time of RP. The most frequently used nsBBs were carvedilol (56.9%) and propranolol (25.4%).

Baseline characteristics stratified according to BB use are detailed in Table 2. Compared with nonusers, a larger proportion of nsBB users were less healthy (Eastern Cooperative Oncology Group [ECOG] performance status >1), older, and comedicated with acetylsalicylic acid, metformin, and/or a statin at the time of RP. There was a tendency toward less favorable prognostic markers among nsBB users compared with nonusers (enrichment of cT3-4, PSA > 20 ng/mL [to convert to micrograms per liter, multiply by 1], International Society of Urological Pathology grade ≥3, and N1 disease).

**Table 2.** Baseline Characteristics of the 11 117 Patients in the Study Cohort^a^

Characteristic	No BB use (n = 9397)	sBB use (n = 1511)	nsBB use (n = 209)	*P* value for sBB use vs no BB use^b^	*P* value for nsBB use vs no BB use^b^
**Patient characteristics**					
Age at RP, median (IQR), y	64.5 (60.1-68.3)	66.7 (62.9-69.7)	66.4 (62.4-69.7)	<.001^c^	<.001^c^
Year of RP	2013 (2010-2014)	2013 (2011-2014)	2012 (2009-2014)	.41^c^	.002^c^
Time from diagnosis to RP, mean (IQR), mo	3.4 (2.3-5.4)	3.4 (2.3-5.6)	3.7 (2.6-5.8)	.36^c^	.04^c^
ECOG performance status					
0-1	6320 (67.3)	995 (65.9)	134 (64.1)	.21^d^	.01^d^
2-4	77 (0.8)	17 (1.1)	5 (2.4)		
Missing	3000 (31.9)	499 (33.0)	70 (33.5)	NA	NA
**Cancer characteristics**					
Clinical T stage					
1-2a	5913 (62.9)	910 (60.2)	122 (58.4)	.047^e^	.70^e^
2b	729 (7.8)	101 (6.7)	23 (11.0)		
2c	965 (10.3)	176 (11.6)	18 (8.6)		
3-4	822 (8.7)	146 (9.7)	19 (9.1)		
Missing data	968 (10.3)	178 (11.8)	27 (12.9)	NA	NA
N stage^f^					
N0/x	8988 (95.6)	1451 (96.0)	198 (94.7)	.30^e^	.16^e^
N1	151 (1.6)	30 (2.0)	6 (2.9)		
Missing	258 (2.7)	30 (2.0)	5 (2.4)		
PSA level, ng/mL					
<10	6005 (63.9)	914 (60.5)	122 (58.4)	.046^e^	.19^e^
10-20	2127 (22.6)	382 (25.3)	52 (24.9)		
>20	447 (4.8)	73 (4.8)	12 (5.7)		
Missing	818 (8.7)	142 (9.4)	23 (11.0)	NA	NA
ISUP grade					
1	1621 (17.3)	257 (17.0)	32 (15.3)	.01^e^	.11^e^
2	4708 (50.1)	701 (46.4)	95 (45.5)		
3	2067 (22.0)	357 (23.6)	54 (25.8)		
4	627 (6.7)	123 (8.1)	19 (9.1)		
5	319 (3.4)	60 (4.0)	7 (3.3)		
Missing	55 (0.6)	13 (0.9)	2 (1.0)	NA	NA
Comedication					
Acetylsalicylic acid	1429 (15.2)	894 (59.2)	102 (48.8)	<.001^d^	<.001^d^
Metformin	335 (3.6)	114 (7.5)	19 (9.1)	<.001^d^	<.001^d^
Statin	2122 (22.6)	942 (62.3)	112 (53.6)	<.001^d^	<.001^d^

Abbreviations: BB, β-blocker; ECOG, Eastern Cooperative Oncology Group; ISUP, International Society of Urological Pathology; NA, not applicable; nsBB, nonselective β-blocker; PSA, prostate-specific antigen; RP, radical prostatectomy; sBB, selective β-blocker.

SI conversion factor: To convert PSA to micrograms per liter, multiply by 1.

^a^ Data are presented as number (percentage) of patients unless otherwise indicated.

^b^ *P* value calculations do not include missing data.

^c^ Mann-Whitney *U* test.

^d^ Pearson χ^2^ test.

^e^ Linear-by-linear association test.

^f^ Nodal stage based on both clinical and pathological data.

### Nonselective β-Blocker Use at RP and Treatment of Recurrence

Median age when receiving treatment of recurrence was 65.8 years (IQR, 62.1-69.4) for nsBB users, 68.2 years (IQR, 64.8-71.0 years) for sBB users, and 66.2 years (IQR, 62.0-69.6 years) for nonusers. The first received treatments for recurrence were radiotherapy (n = 1252), hormonal therapy (n = 352), and chemotherapy (n = 5); 13 patients were registered as having prostate cancer–specific death without prior treatment of recurrence.

Cox proportional hazards regression analysis, using imputed data for missing covariates, showed that nsBB use at the time of surgery was significantly associated with less treatment for recurrence (adjusted hazard ratio [aHR], 0.64; 95% CI, 0.42-0.96; *P* = .03) (Table 3). The analysis was adjusted for age, clinical T stage, International Society of Urological Pathology grade, PSA level, N stage, ECOG performance status, year of surgery, and time from initial diagnosis to surgery (Table 4). A corresponding complete case analysis (n = 7147) showed a similar and significant association between nsBB use and treatment for recurrence (aHR, 0.51; 95% CI, 0.28-0.93; *P* = .03). Use of sBB at the time of surgery was not associated with treatment for recurrence in the imputed data set (aHR, 0.96; 95% CI, 0.84-1.11; *P* = .62) or the complete case analysis (aHR, 0.94; 95% CI, 0.78-1.14; *P* = .53).

**Table 3.** Multivariable Cox Proportional Hazards Regression Analysis of BB Exposure and Treatment for Recurrence^a^

Exposure	Adjusted hazard ratio (95% CI)	*P* value
Imputed data set^b^		
No BB use	1 [Reference]	NA
sBB use	0.96 (0.84-1.11)	.62
nsBB use	0.64 (0.42-0.96)	.03
Complete cases^c^		
No BB use	1 [Reference]	NA
sBB use	0.94 (0.78-1.14)	.53
nsBB use	0.51 (0.28-0.93)	.03

Abbreviations: BB, β-blocker; NA, not applicable; nsBB, nonselective β-blocker; sBB, selective β-blocker.

^a^ The multivariate model was adjusted for clinical T stage, pathological or clinical N stage, International Society of Urological Pathology grade, prostate-specific antigen level, and baseline patient characteristics (age at radical prostatectomy, time from diagnosis to radical prostatectomy, year of radical prostatectomy, and Eastern Cooperative Oncology Group performance status).

^b^ Pooled results from 50 imputations (11 117 patients and 1622 events).

^c^ Complete case analysis (7147 cases and 939 events).

**Table 4.** Hazard Ratios of Covariates Included in Multivariate Analysis of Imputed Data

Variable	Adjusted hazard ratio (95% CI)	*P* value
Age at RP	1.00 (0.99-1.01)	.96
Year of RP	0.93 (0.90-0.96)	<.001
Time from diagnosis to RP	1.00 (1.00-1.00)	.003
ECOG performance status		
1-2	1 [Reference]	NA
2-4	1.02 (0.69-1.52)	.91
Clinical T stage		
T1-2a	1 [Reference]	NA
T2b	1.16 (.97-1.38)	.10
T2c	.99 (.84-1.16)	.86
T3-4	1.37 (1.15-1.64)	.001
N stage^a^		
N0/x	1 [Reference]	NA
N1	2.77 (2.18-3.58)	<.001
ISUP grade		
1	1 [Reference]	NA
2	1.95 (1.58-2.40)	<.001
3	4.75 (3.83-5.88)	<.001
4	6.89 (5.40-8.80)	<.001
5	9.30 (7.17-12.05)	<.001
PSA level, ng/mL		
<10	1 [Reference]	NA
10-20	1.44 (1.28-1.61)	<.001
>20	1.83 (1.52-2.21)	<.001

Abbreviations: ECOG, Eastern Cooperative Oncology Group; ISUP, International Society of Urological Pathology; NA, not applicable; PSA, prostate-specific antigen; RP, radical prostatectomy.

SI conversion factor: To convert PSA to micrograms per liter, multiply by 1.

^a^ On the basis of clinical and pathological findings.

### Sensitivity Analyses

To test for potential bias introduced by excluding patients receiving treatment within 6 months after surgery, 2 sensitivity analyses with relaxed exclusion criteria were performed: no exclusion for early treatment after RP (n = 12 298) and exclusion of patients receiving treatment within the first 3 months after RP (n = 11 886). Clinical characteristics for these additional patients are presented in eTables 3 and 4 in the Supplement. In the complete case analyses, nsBB use was significantly associated with less treatment for recurrence, both when excluding no patients for early treatment (aHR, 61; 95% CI, 0.40-.94; *P* = .03) (eTable 5 in the Supplement) and when excluding only patients receiving treatment within the first 3 months (aHR, 0.57; 95% CI, 0.35-.91; *P* = .02) (eTable 6 in the Supplement). No associations were observed in the imputed data set (eTables 5 and 6 in the Supplement).

Because the ECOG performance status differed significantly between nsBB users and nonusers as well as between sBB users and nonusers, subanalyses that included only patients with an ECOG performance status of 0 were performed, also including an analysis with relaxed inclusion criteria to allow inclusion of patients with early treatment for recurrence or surgical failure 3 to 6 months after RP. These analyses yielded results similar to the corresponding analyses of patients with all ECOG performance statuses (eTables 7 and 8 in the Supplement).

During follow-up, 330 men (3.0%) died. The proportion of men registered as dead from causes other than prostate cancer (n = 286) was significantly higher in both nsBB users (11 [5.3%]) and sBB users (54 [3.6%]) compared with nonusers of BBs (221 [2.4%]). Only 30 (10.5%) of these patients received treatment for recurrence before death. A complete case competing risk analysis was performed to address the potential bias resulting from death that occurred before sufficient time to receive treatment for recurrence. Adjusted HRs for nsBB use were similar to the main findings (aHR, 0.52; 95% CI, 0.28-0.94; *P* = .03).

Among nonusers of nsBB at RP, 202 men with previous use of nsBB were identified. No association was found between previous use and treatment for recurrence (eTable 10 and eFigure in the Supplement).

Sensitivity analysis stratified on duration of nsBB use before RP (<6 months [short-term use; n = 11] and ≥6 months [extended use; n = 198]) showed a statistically significant association with treatment for recurrence only for extended use (aHR, 0.64; 95% CI, 0.42-0.96; *P* = .03). However, the aHR for short-term use was of a similar size (aHR, 0.68; 95% CI, 0.09-4.68; *P* = .70) (eTable 11 in the Supplement). Adjusting for concurrent use of acetylsalicylic acid, metformin, and statins at time of RP did not influence our findings (eTable 12 in the Supplement).

## Discussion

The main finding in this cohort study is that incidental use of an nsBB at the time of RP was significantly associated with less initiation of treatment for prostate cancer recurrence. No such association was observed among users of sBBs.

Surgery is a major stress-inducing event, activating the fight-or-flight response. In response to surgery, a multistep wound-healing process is induced. Many similarities exist between the molecular mechanisms involved in wound healing and cancer progression (ie, migration, immune cell infiltration, angiogenesis, and cellular linage plasticity).^34^ The β_2_-AR has been identified as the key adrenergic receptor over β_1_-AR, promoting catecholamine-induced tumorigenesis.^35^ In addition, an increasing amount of preclinical evidence supports that perioperative use of nsBBs has a beneficial effect on oncological outcome.^6,7,8^ This finding could explain why an association with prostate cancer progression was only observed for nsBBs, which act on both β_1_- and β_2_-ARs, and not for sBBs, which target mainly β_1_-AR. Indeed, randomized clinical trials have been performed assessing perioperative use of nsBBs alone, in breast and ovarian cancer, and in combination with cyclooxygenase 2 inhibition in colorectal cancer.^20,36,37^

Despite the accumulated evidence supporting nsBB use as beneficial to target the perioperative surgical stress response,^6,7,8,11,12,20,36,37^ other studies^24,38^ have challenged this concept. In a retrospective cohort study by Musselman et al,^38^ no associations between perioperative use of BBs or nsBB and overall survival or cancer-specific survival in patients who had undergone surgery for breast, lung, and colorectal cancer were reported. However, the inability to match cases based on cancer stage may have contributed to the null findings of that study. Similarly, in a recent publication^24^ that assessed BB use in patients with prostate cancer treated with androgen deprivation therapy, no significant association was found between BB use and oncological outcomes. However, although users of nsBB were identified, they were not separately analyzed. Furthermore, only a small proportion had previously undergone RP; hence, no assessment of any associations between perioperative BB use and progression was performed.

We acknowledge that starting follow-up 6 months after RP, excluding patients receiving treatment for recurrence or lost to follow-up during the first 6 months, introduces a selection bias by not including a proportion of patients with more aggressive disease at RP. However, because we did not have information on postoperative PSA values or surgical margins, starting follow-up at 6 months was decided a priori. The rationale was to achieve a cohort of patients not in need of immediate additional treatment because of surgical failure (locoregional residual disease) or unrecognized metastatic disease at time of RP, factors unlikely to be directly influenced by nsBBs, but potentially influenced by unregistered confounders. For example, obesity is known to lead to more challenging surgery and potentially higher rates of surgical failure (eg, positive surgical margins).^39,40^ Because BB use is associated with obesity and weight gain,^41,42^ obesity may partially explain the lack of association between nsBB use and less initiation of treatment for recurrence in the imputed data set when patients receiving treatments during the first 6 months after RP are included. The use of an nsBB itself at the time of surgery is unlikely to influence the risk of surgical failure.

All-cause mortality was higher among BB users than nonusers. Nevertheless, the result of the competing risk analysis was similar to the main analysis, suggesting that the association between nsBB use at RP and progression-free survival is not influenced by mortality potentially precluding treatment for prostate cancer recurrence.

Statins, metformin, and acetylsalicylic acid have previously been reported to affect oncological outcome in patients with prostate cancer.^43^ Although the indications for these drugs and BBs are largely overlapping, adjusting for use of these drugs did not influence our findings, indicating that the association between nsBB use and progression was not confounded by these factors.

The introduction of early salvage treatment in recent years makes the decision basis for early (within 6 months) and late (after 6 months) treatment of recurrence less predictable,^44^ especially without knowledge of post-RP PSA values and surgical margins. However, because our cohort consisted of patients operated on from 2008 to 2015, we do not regard this as a significant issue in our cohort.

### Limitations

This study has some limitations. Because of missing data, only 7147 patients (64.3%) were eligible for complete case analysis, introducing a potential bias attributable to unrecognized structurally missing data. However, the prospective nature of and mandatory reporting to the national registries make it likely that data are missing completely at random. Furthermore, we believe that performing multiple imputation makes the potential bias of unrecognized structurally missing data less likely to influence our findings.

An important limitation is the lack of postoperative PSA values and hence the inability to identify biochemical recurrence. Treatment for recurrence was therefore used as a surrogate end point, but eligibility for this treatment is influenced by comorbidities and life expectancy, both of which (illustrated by ECOG performance status and deaths not related to prostate cancer) differed significantly between users of nsBBs and nonusers. However, limiting analyses to the healthiest individuals at time of RP (ECOG performance status of 0) yielded results similar to our main finings. In addition, the lack of any association between use of sBBs and treatment for progression suggests that the results are not heavily influenced by these potential confounders; because sBBs and nsBBs are used for largely overlapping indications, these factors could be expected to be similar between the 2 groups.

We acknowledge the potential residual confounding from background variables associated with cancer recurrence, such as socioeconomic status, race and ethnicity, and smoking habits. Unfortunately, this information was not available from the included registries. However, again because of the overlapping indications of nsBBs and sBBs, we believe our findings are unlikely to be fully explained by residual confounding.

Although the aim was to assess the use of nsBBs at the time of surgery, patients identified as nsBB users at RP had used the medication for a median of more than 4 years. Because few nsBB users had used nsBB for less than 6 months before RP, stratifying nsBB use on prior use for less than 6 months or 6 months or more was underpowered to determine whether the association was with current vs with prior use. Still, the similar aHR values for the 2 nsBB user groups (0.68 vs 0.64) at least suggest that the association is similar regardless of duration of use. Furthermore, the lack of any association with previous nsBB use suggests that current use at least is of importance.

## Conclusions

To our knowledge, this is the first study looking specifically at an association between use of nsBBs at the time of surgery and recurrence-related treatment. With the high volumes of RPs being performed, even a small delay in the need for further hormonal therapy and radiotherapy after surgery would justify an interventional study to identify a potential causality between nsBB use and prostate cancer recurrence.

## Notes

Supplement. **eTable 1.** Filled Prescriptions Within 100 Days Prior to Surgery; β-Blockers and Hormonal Therapy **eTable 2.** Multiple Imputation: Imputed and Dependent Variables **eTable 3.** Baseline Characteristics—All Excluded Patients (N = 1181) **eTable 4.** Baseline Characteristics—Patients Excluded; Follow-up 3 to 6 Months (n = 749) **eTable 5.** Multivariate Cox Analysis, Relaxed Exclusion Criteria; No Exclusion (n = 12 298) **eTable 6.** Multivariate Cox Analysis, Relaxed Exclusion Criteria; Excluding Events <3 Months (n = 11 886) **eTable 7.** Multivariate Cox Analysis, Healthiest Patients (ECOG 0) **eTable 8.** Multivariate Cox Analysis, Relaxed Exclusion Criteria, Excluding Events <3 Months, Healthiest; ECOG = 0 **eTable 9.** Duration of Use of nsBB **eTable 10.** Multivariate Cox Analysis; Previous Use of nsBB vs Current Use of nsBB **eTable 11.** Multivariate Cox Analysis; Stratified on Duration of nsBB Use Before Radical Prostatectomy **eTable 12.** Multivariate Cox Analysis; Comedication of Acetylsalicylic Acid (ASA), Metformin, and Statin **eFigure.** Flowchart Illustrating the Definition of Drug Users
