# Supplementary material for: Association of β-Blocker Use at Time of Radical Prostatectomy With Rate of Treatment for Prostate Cancer Recurrence
Source: JAMA Netw Open. 2022 Jan 26;5(1):e2145230. doi: 10.1001/jamanetworkopen.2021.45230 (PMC8792886; doi:10.1001/jamanetworkopen.2021.45230)
Supplement: Supplement. — eTable 1. Filled Prescriptions Within 100 Days Prior to Surgery; β-Blockers and Hormonal Therapy eTable 2. Multiple Imputation: Imputed and Dependent Variables eTable 3. Baseline Characteristics—All Excluded Patients (N = 1181) eTable 4. Baseline Characteristics—Patients Excluded; Follow-up 3 to 6 Months (n = 749) eTable 5. Multivariate Cox Analysis, Relaxed Exclusion Criteria; No Exclusion (n = 12 298) eTable 6. Multivariate Cox Analysis, Relaxed Exclusion Criteria; Excluding Events <3 Months (n = 11 886) eTable 7. Multivariate Cox Analysis, Healthiest Patients (ECOG 0) eTable 8. Multivariate Cox Analysis, Relaxed Exclusion Criteria, Excluding Events <3 Months, Healthiest; ECOG = 0 eTable 9. Duration of Use of nsBB eTable 10. Multivariate Cox Analysis; Previous Use of nsBB vs Current Use of nsBB eTable 11. Multivariate Cox Analysis; Stratified on Duration of nsBB Use Before Radical Prostatectomy eTable 12. Multivariate Cox Analysis; Comedication of Acetylsalicylic Acid (ASA), Metformin, and Statin eFigure. Flowchart Illustrating the Definition of Drug Users [file jamanetwopen-e2145230-s001.pdf]

Supplementary Online Content

Sivanesan S, Taskén KA, Grytli HH. Association of β-blocker use at time of radical prostatectomy with rate of treatment for prostate cancer recurrence. *JAMA Netw Open*. 2022;5(1):e2145230. doi:10.1001/jamanetworkopen.2021.45230

eTable 1. Filled Prescriptions Within 100 Days Prior to Surgery; β-Blockers and Hormonal Therapy

eTable 2. Multiple Imputation: Imputed and Dependent Variables

eTable 3. Baseline Characteristics—All Excluded Patients (N = 1181)

eTable 4. Baseline Characteristics—Patients Excluded; Follow-up 3 to 6 Months (n = 749)

eTable 5. Multivariate Cox Analysis, Relaxed Exclusion Criteria; No Exclusion (n = 12 298)

eTable 6. Multivariate Cox Analysis, Relaxed Exclusion Criteria; Excluding Events < 3 Months (n = 11 886)

eTable 7. Multivariate Cox Analysis, Healthiest Patients (ECOG 0)

eTable 8. Multivariate Cox Analysis, Relaxed Exclusion Criteria, Excluding Events < 3 Months, Healthiest; ECOG = 0

eTable 9. Duration of Use of nsBB

eTable 10. Multivariate Cox Analysis; Previous Use of nsBB vs Current Use of nsBB

eTable 11. Multivariate Cox Analysis; Stratified on Duration of nsBB Use Before Radical Prostatectomy

eTable 12. Multivariate Cox Analysis; Comedication of Acetylsalicylic Acid (ASA), Metformin, and Statin

eFigure. Flowchart Illustrating the Definition of Drug Users

This supplementary material has been provided by the authors to give readers additional information about their work.

eTable 1. Filled prescriptions within 100 days prior to surgery; beta-blockers and hormonal therapy

	ATC code:	Generic name:	N (%):
**Non-selective beta-blocker**	C07AG02	Carvedilol	119 (56.9)
	C07AA05	Propranolol	53 (25.4)
	C07AA07	Sotalol	29 (13.9)
	C07AG01	Labetalol	4 (1.9)
	C07AA06	Timolol	3 (1.4)
	C07AA03	Pindolol	1 (0.5)
**Selective beta-blocker**	C07AB02	Metoprolol	1244 (82.3)
	C07AB03	Atenolol	174 (11.5)
	C07AB07	Bisprolol	56 (3.7)
	C07BB07	Bisprolol + thiazide	37 (2.5)
**Hormonal therapy**	L02BB03	Biculatamide	655 (93.8)
	L02BX02	Degarelix	28 (4.0)
	L02AE02	Leuprorelin	13 (1.9)
	L02AE03	Goserelin	2 (0.3)

eTable 2. Multiple Imputation: imputed and dependent variables

Variable:	Role:	Imputation type :	Missingness: N (%):
ISUP grade	Imputed and dependent	Logistic regression	98 (0.8)
Metastasis[a]	Imputed and dependent	Logistic regression	352 (2.9)
PSA interval	Imputed and dependent	Logistic regression	1110 (9.0)
cT stage	Imputed and dependent	Logistic regression	1333 (10.8)
ECOG	Imputed and dependent	Logistic regression	3916 (31.8)
Type of surgery[b]	Imputed and dependent	Logistic regression	5064 (41.1)
pT stage	Imputed and dependent	Logistic regression	5183 (42.1)
pN stage	Imputed and dependent	Logistic regression	7389 (59.2)
PLND (yes/no)	Imputed and dependent	Logistic regression	8390 (68.1)
Nerve sparing surgery (yes/no)	Imputed and dependent	Logistic regression	8576 (69.7)
Prostate volume (categorized)[c]	Imputed and dependent	Logistic regression	10278 (83.5)
nsBB, sBB, or non-user[d]	Dependent		
ASA[d]	Dependent		
Metformin[d]	Dependent		
Statin[d]	Dependent		
Recurrence (Yes/No)	Dependent		
Endpoint due to hormonal therapy	Dependent		
Endpoint due to chemotherapy	Dependent		
Cause of death	Dependent		
ICD03 morphology	Dependent	*Not imputed, no missing values*	
Year of birth	Dependent		
Year of diagnosis	Dependent		
Age at RP	Dependent		
Time from diagnosis to RP (days)	Dependent		
Year of RP	Dependent		
Time of RP	Dependent		
Follow-up time (days)	Dependent		

eTable 2 footnote:

Abbreviations: PLND, Pelvic lymph node dissection; ASA, acetylsalicylic acid; nsBB, non-selective beta-blocker; sBB, selective beta-blocker; RP, radical prostatectomy; ECOG, Eastern Cooperative Oncology Group performance status
Imputation was performed on SPSS v 26.0, with fully conditional specification (FCS) imputation.
[a] Based on disease stage reported to the Cancer registry of Norway, including both clinical and/or pathological data, grouping patients into: N0M0 <T3; T3 ;T4; N1; M1a/b/c/+ .
[b] Dichotomized into mini-invasive (robotic and laparoscopic RP) and open (perineal or retropubic RP)
[c] Five categories: < 20, 20-40, 40-60, 60-80, and >80 ml.
[d] At time of surgery.

**eTable 3.** Baseline characteristics - all excluded patients (n=1181)					
	**No BB** (n=994)	**sBB** (n=163)	**nsBB** (n=24)	**P-vaule**[a]	**P-value**[b]
**Patient characteristics:**					
Age at RP	65.4 (60.7-69.3)	66.8 (64.0-70.1)	65.4 (63.0-67.8)	<.001[c]	.98[c]
RP year	2013 (2011-2015)	2013 (2011-2015)	2012 (2010-2014)	.27[c]	.12[c]
Time to RP (months)[d]	2.9 (2.1-4.7)	3.2 (2.1-5.7)	4.1 (2.7-5.6)	.09[c]	.07[c]
ECOG					
0-1	704 (70.8)	115 (70.6)	12 (50)	.09[e]	.70[e]
2-4	9 (0.9)	4 (2.5)	0		
Missing	281 (28.3)	44 (27.0)	12 (50)	[f]	[f]
**Cancer characteristics:**					
Clinical Tumor stage					
1-2a	465 (46.8)	62 (38.0)	12 (50)	.02[g]	.08[g]
2b	92 (9.3)	12 (7.4)	0		
2c	96 (9.7)	17 (10.4)	2 (8.3)		
3-4	214 (21.5)	46 (28.2)	5 (20.8)		
Missing	127 (12.8)	26 (16.0)	5	[f]	[f]
Nodal stage[h]					
N0/x	679 (68.3)	114 (69.9)	16 (66.7)	.45[e]	.64[e]
N1	270 (27.2)	39 (23.9)	5 (20.8)		
Missing	45 (4.5)	10 (6.1)	3 (12.5)	[f]	[f]
PSA interval					
<10	363 (36.5)	71 (43.6)	9 (37.5)	.16[g]	.89[g]
10-20	357 (35.9)	44 (27.0)	7 (29.2)		
>20	172 (17.3)	27 (16.6)	5 (20.8)		
Missing	102 (10.3)	21 (12.9)	3 (12.5)	[f]	[f]
ISUP grade group					
1	33 (3.3)	5 (3.1)	2 (8.3)	.86[gh]	.07[gh]
2	263 (26.5)	40 (24.5)	8 (33.3)		
3	264 (26.6)	41 (25.2)	8 (33.3)		
4	175 (17.6)	43 (26.4)	1 (4.2)		
5	238 (23.9)	28 (17.2)	4 (16.7)		
Missing	21 (2.1)	6 (3.7)	1 (4.2)	[f]	[f]
**Co-medication**					
Acetylsalicylic acid	124 (12.5)	93 (57.1)	11 (45.8)	<.001[e]	<.001[e]
Metformin	44 (4.4)	18 (11.0)	2 (8.3)	.001[e]	.36[e]
Statin	211 (21.2)	103 (63.2)	15 (62.5)	<.001[e]	<.001[e]

eTable 3 Footnote:
Baseline characteristics of all patients excluded due to inclusion due to follow-up <six months after radical prostatectomy (RP).

eTable 3 Footnote continued:
Estimates given as median (quartile 1, quartile 3) or frequency (percentage).
Abbreviations: nsBB, non-selective β-blocker users; sBB, selective β-blocker users; No BB, non-users of β-blockers; RP, Radical Prostatectomy; ECOG, Eastern Cooperative Oncology Group performance status; cT, clinical tumor stage; N, Nodal stage
[a]Users of sBB vs non-BB users. [b]Users of nsBB vs non-BB users. [c]Mann-Whitney U test. [d]Months from diagnosis to RP; [e]Pearson's chi². [f]P-value calculations do not include missing data; [g]Linear-by-linear association test; [h]Nodal stage based on both clinical and pathological data.

**eTable 4.** Baseline characteristics - patients excluded; follow-up three to six months (n=749)					
	**No BB** (n=614)	**sBB** (n=118)	**nsBB** (n=17)	**P-value**[a]	**P-value**[b]
**Patient characteristics**					
Age at RP	65.7 (60.1-69.8)	66.8 (64.0-70.0)	65.8 (64.0-67.7)	.02[c]	.85[c]
RP year	2013 (2011-2015)	2013 (2011-2015)	2011 (2009-2013)	.12[c]	.04[c]
Time to RP (months)[d]	2.9 (2.1-4.6)	3.2 (2.1-5.4)	4.5(3.0-5.6)	.16[c]	.09[c]
ECOG					
0-1	432 (70.4)	85(72.0)	7(41.2)	.34[e]	.72[e]
2-4	8 (1.3)	3(2.5)	0		
Missing	174 (28.3)	30(25.4)	10(58.8)	[f]	[f]
**Cancer characteristics:**					
Clinical Tumor stage					
1-2a	302 (49.2)	44(37.3)	7(41.2)	.02[g]	.55[g]
2b	50 (8.1)	8(6.8)	0		
2c	61 (9.9)	12(10.2)	2(11.8)		
3-4	125 (20.4)	33(28.0)	4(23.5)		
Missing	76 (12.4)	21(17.8)	4(23.5)	[f]	[f]
Nodal stage[h]					
N0/x	482 (78.5)	97 (82.2)	13 (76.5)	.38[e]	.57[e]
N1	114 (18.6)	18 (15.3)	2 (11.8)		
Missing	18 (2.9)	3 (2.5)	2 (11.8)	[f]	[f]
PSA interval					
<10	217 (35.3)	51 (43.2)	3 (17.6)	.24[g]	.09[g]
10-20	229 (37.3)	29 (24.6)	6(35.3)		
>20	106 (17.3)	21 (17.8)	5 (29.4)		
Missing	62 (10.1)	17 (14.4)	3 (17.6)	[f]	[f]
ISUP grade group					
1	26 (4.2)	5 (4.2)	2 (11.8)	.55[g]	0.42[g]
2	169 (27.5)	29 (24.6)	5 (29.4)		
3	177 (28.8)	30 (25.4)	5 (29.4)		
4	119 (19.4)	33 (28.0)	1 (5.9)		
5	123 (20.0)	21 (17.8)	4 (23.5)		
Missing data	0	0	0	[f]	[f]
**Co-medication**					
Acetylsalicylic acid	78 (12.7)	71 (60.2)	8 (47.1)	<.001[e]	<.001[e]
Metformin	24 (3.9)	14 (11.9)	2 (11.8)	<.001[e]	0.11[e]
Statin	134 (21.8)	78 (66.1)	11 (64.7)	<.001[e]	<.001[e]

eTable4 Footnote:
Baseline characteristics of patients excluded with a follow up from three months, but <six months.
Estimates given as median (quartile 1, quartile 3) or frequency (percentage).

eTable4 Footnote continured:
Abbreviations: nsBB, non-selective β-blocker users; sBB, selective β-blocker users; No BB, non-users of β-blockers; RP, Radical Prostatectomy; ECOG, Eastern Cooperative Oncology Group performance status; cT, clinical tumor stage; N, Nodal stage
[a]Users of sBB vs non-BB users. [b]Users of nsBB vs non-BB users. [c]Mann-Whitney U test. [d]Months from diagnosis to RP; [e]Pearson's chi². [f]P-value calculations do not include missing data; [g]Linear-by-linear association test; [h]Nodal stage based on both clinical and pathological data.

**eTable 5.** Multivariate Cox analysis[a], relaxed exclusion criteria; no exclusion (n=12298)			
	**Exposure:**	**Hazard ratio (95% CI)**	**P-value**
Imputed[b]	non-BB use	*Reference*	
	sBB user	.97 (.87 – 1.09)	.66
	nsBB user	.79 (.59 – 1.07)	.13
Complete cases[c]	non-BB use	*Reference*	
	sBB user	.96 (.83 – 1.11)	.58
	nsBB user	.61 (.40 – .94)	.03

eTable 5 Footnote:
Analysis of associations between β-blocker exposure at radical prostatectomy (RP) and treatment for recurrence in cohort with relaxed exclusion criteria – no exclusions due to early treatment of recurrence after RP (total n of 12298).

Abbreviations: nsBB, non-selective β-blocker; sBB, selective β-blocker; nonBB, non β-blockers.
[a]Multivariate cox proportional hazard model adjusted for cT stage, nodal-stage, ISUP grade group, PSA interval and basic patients characteristics (age at RP, time from diagnosis to RP, year of RP and ECOG).
[b]Pooled results from 50 imputations; 12298, 2682 events. [c]Complete case analysis, 7921 cases, 1626 events.

**eTable 6.** Multivariate Cox analysis[a], relaxed exclusion criteria; excluding events < three months (n=11886)			
	**Exposure:**	**Hazard ratio (95% CI)**	**P-value**
Imputed[b]	non-BB users	*Reference*	
	sBB user	1.01 (.90 -1.14)	.90
	nsBB user	.77 (.56 -1.06)	.11
Complete cases[c]	*non-BB users*	*Reference*	
	sBB user	1.02 (.87 - 1.18)	.83
	nsBB user	.57 (.35 - .91)	.02

eTable 6 Footnote:
Analysis of associations between β-blocker exposure at radical prostatectomy (RP) and treatment for recurrence in cohort with relaxed exclusions criteria – only excluding those who received treatment for cancer within three months after RP (total n of 11886).

Abbreviations: nsBB, non-selective β-blocker; sBB, selective β-blocker; nonBB, non β-blockers.
[a]Multivariate cox proportional hazard model adjusted for cT stage, nodal-stage, ISUP grade group, PSA interval and basic patients characteristics (age at RP, time from diagnosis to RP, year of RP and ECOG).
[b]Pooled results from 50 imputations; 11866, 2318 events.
[c]Complete case analysis, 7652 cases, 1399 events

**eTable 7.** Multivariate Cox analysis[a], healthiest patients (ECOG 0)			
	**Exposure:**	**Hazard ratio (95% CI)**	**P-value**
Imputed[b]	non-BB users	*Reference*	
	sBB user	.93 (.77 - 1.13)	.47
	nsBB user	.55 (.31 - .99)	.05[c]
Complete cases[d]	non-BB users	*Reference*	
	sBB user	.93 (.75 - 1.14)	.46
	nsBB user	.51 (.27 - .95)	.04

eTable 7 Footnote:
Analysis of associations between BB exposure at radical prostatectomy and treatment for recurrence among the healthiest patients (ECOG =0) in our study cohort.

Abbreviations: nsBB, non-selective β-blocker; sBB, selective β-blocker; nonBB, non β-blockers.
[a]Multivariate cox proportional hazard model adjusted for cT stage, nodal-stage, ISUP grade group, PSA interval and basic patients characteristics (age at RP, time from diagnosis to RP and year of RP). [b]Pooled results from 50 imputations; 8457, 1115 events. [c]Rounded up to .05 from actual value of ≥ .045. [d]Complete case analysis, 6559 cases, 865 events

**eTable 8.** Multivariate Cox analysis[a], relaxed exclusion criteria, excluding events < three months, healthiest; ECOG=0			
	**Exposure:**	**Hazard ratio (95% CI)**	**P-value**
Imputed[b]	non-BB users	*Reference*	
	sBB user	1.00 (.86 - 1.17)	.95
	nsBB user	.62 (.37 - 1.03)	.07
Complete cases[c]	non-BB users	*Reference*	
	sBB user	1.01 (.86 - 1.19)	.88
	nsBB user	.51 (.30 - .87)	.01

eTable 8 Footnote:
Analysis of associations between BB exposure at RP and treatment for recurrence among the healthiest patients (ECOG =0) in cohort with relaxed exclusions criteria – only excluding those who received treatment for cancer within three months after radical prostatectomy.

Abbreviations: nsBB, non-selective β-blocker; sBB, selective β-blocker; nonBB, non β-blockers.
[a]Multivariate cox proportional hazard model adjusted for cT stage, nodal-stage, ISUP grade group, PSA interval and basic patients characteristics (age at RP, time from diagnosis to RP and year of RP)
[b]Pooled results from 50 imputations; 8987, 1601 events. [c]Complete case analysis, 7011 cases, 1277 events

**eTable 9 .** Duration of use of nsBB		
**nsBB users at RP; length of use:**	**Number of users** n (%)	**Duration in days prior to RP** Median (IQR)
**Main cohort (follow up from 6m/183d after RP):**		
[a]Known duration:	145 (69.4)	1516 (848-2476)
[b]Duration equals or is longer than:	64 (30.6)	2760 (1986-3243)
**Whole cohort (follow up from RP):**		
[a]Known duration:	161 (69.1)	1573 (937-2600)
[b]Duration equals or is longer than:	72 (30.9)	2681 (1888-3239)
**Cohort with events (first 6m/182d after RP):**		
[a]Known duration:	16 (66.7)	2661 (1527-3083)
[b]Duration equals or is longer than:	8 (33.3)	1958 (1498-3050)
**Prior nsBB users; length of use:**	**Number of users** n (%)	**Duration in days prior to RP** Median (IQR)
**Main cohort (follow up from 6m/183d after RP):**		
[a]Known duration:	157 (77.7)	1965 (1165-3055)
[b]Duration equals or is longer than:	45 (22.3)	3004 (2478-3942)
**Whole cohort (follow up from RP):**		
[a]Known duration:	170 (76.2)	1971 (1171-3053)
[b]Duration equals or is longer than:	53 (23.8)	3141 (2636-4099)
**Cohort with events (first 6m/182d after RP):**		
[a]Known duration:	13 (61.9)	2048 (1601-3183)
[b]Duration equals or is longer than:	8 (38.1)	4356 (4022-4563)

eTable 9 Footnote:
Overview of nsBB use in the study cohort (n=11117); nsBB (present) users at RP and previous nsBB users (considered non-users at RP).

Abbreviations: nsBB, non-selective β-blocker; RP, radical prostatectomy.
Estimates given as median (quartile 1, quartile 3) or frequency (percentage).
[a]Users that have filled a prescription ≥ 100 days after the first Norwegian Prescription Database entry (Jan 1st, 2004).
[b]Users that have filled a prescription < 100 days after the first Norwegian Prescription Database entry (Jan 1st, 2004).
(100 days is used, as repeated prescription medications are usually given in quantities sufficient for 3 months use)
.

**eTable 10.** Multivariate Cox analysis[a]**;** previous use of nsBB vs current use of nsBB			
	**Exposure:**	**Hazard ratio (95% CI)**	**P-value**
Imputed[b]	nonBB[c]	*Reference*	
	previous nsBB user[d]	.96 (.65-1.42)	.83
	current nsBB user[e]	.64 (.43-.96)	.03
Complete cases[f]	nonBB[c]	*Reference*	
	previous nsBB user[d]	1.16 (.74-1.81)	.51
	current nsBB user[e]	0.52 (.29-.94)	.03

eTable 10 Footnote:
Analysis of associations between previous nsBB use (not considered current user at RP) and current use of a nsBB at RP, and treatment for recurrence.

Abbreviations: nsBB, non-selective β-blocker; sBB, selective β-blocker; nonBB, no β-blocker
[a]Multivariate cox proportional hazard model adjusted for cT stage, nodal-stage, ISUP grade group, PSA interval and basic patients characteristics (age at RP, time from diagnosis to RP, year of RP and ECOG)
[b]Pooled results from 50 imputations; 9681 cases, 1408 events
[c]No previous nsBB use, and not considered sBB or nsBB user at RP.
[d]Prior use of nsBB identified, but not considered users at RP(i.e. no filled prescription of a nsBB within 100d prior to RP); this group includes 75 patients considered users of sBB at RP and 127 patients with no use of any BB at RP.
[e]Current users of a nsBB at RP (i.e. the 209 patients that had filled a prescription of a nsBB within 100d of RP).
[f]Complete case analysis, 6056 cases, 824 events

eTable 11. Multivariate Cox analysis[a]; stratified on duration of nsBB use before radical prostatectomy

	**Exposure:**	**Hazard ratio(95% CI)**	**P-value**
Imputed[b]	non-user[c]	*Reference*	
	≥ six months use[d]	.64(.42-.96)	.03
	<six months use[e]	.68(.09-4.68)	.70
Complete cases[f]	non-user[c]	*Reference*	
	≥ six months use[d]	.49 (.27-.92)	.03
	<six months use[e]	1.00 (.14-7.14)	.99

eTable 11 Footnote:
Analyses of associations between treatment for recurrence and duration of nsBB use (< vs ≥ six months) prior to after radical prostatectomy.

Abbreviations: nsBB, non-selective β-blocker; RP, radical prostatectomy
[a]Multivariate cox proportional hazard model adjusted for cT stage, nodal-stage, ISUP grade group, PSA interval and basic patients characteristics (age at RP, time from diagnosis to RP, year of RP and ECOG)
[b]Pooled results from 50 imputations; 11117 cases, 1622 events
[c]Non use of nsBBs and selective BBs at time of RP
[d]Users of nsBB with duration of use ≥6m prior to RP
[e]Users of nsBB with duration of use <6m prior to RP
[f]Complete case analysis, 7147 cases, 939 events

eTable 12. Multivariate Cox analysis[a]; co-medication of acetylsalicylic acid (ASA), metformin and statin.

		Exposure:	Hazard ratio (95% CI):	P-value
**Co-medication: BBs, acetylsalicylic acid (ASA), metformin, statin[b]**				
Imputed[c]	BBs	non-BB use	*Reference*	
		sBB user	.96 (.82 – 1.12)	.63
		nsBB user	.64 (.42 – .96)	.03
	ASA	non-ASA user	*Reference*	
		ASA user	1.01(.88 – 1.16)	.92
	Metformin	non-metformin user	*Reference*	
		metformin user	.99 (.77 – 1.26)	.90
	Statin	non-statin user	*Reference*	
		statin user	1.00 (.88 – 1.14)	1.00*
Complete cases[d]	BBs	non-BB use	*Reference*	
		sBB user	.91 (.74 – 1.12)	.38
		nsBB user	.50 (.28 – .91)	.02
	ASA	non-ASA user	*Reference*	
		ASA user	1.09 (.91 – 1.31)	.37
	Metformin	non-metformin user	*Reference*	
		metformin user	0.95 (.70 – 1.30)	.76
	Statin	non-statin user	*Reference*	
		statin user	0.99 (.83 – 1.17)	.87
**Co-medications: Acetylsalicylic acid (ASA) only[e]**				
Imputed[c]		non-ASA use	*Reference*	
		ASA user	0.98 (0.87 – 1.11)	.79
Complete cases[d]		non-ASA use	*Reference*	
		ASA user	1.03 (0.89 – 1.20)	.69
**Co-medication: Metformin only[f]**				
Imputed[c]		non-ASA use	*Reference*	
		ASA user	0.98 (0.77 – 1.25)	.86
Complete cases[d]		non-ASA use	*Reference*	
		ASA user	0.95 (0.71 – 1.29)	.76
**Co-medication adjusted for: Statin only[g]**				
Imputed[c]		non-ASA use	*Reference*	
		ASA user	0.99 (0.88 – 1.10)	.79
Complete cases[d]		non-ASA use	*Reference*	
		ASA user	0.99 (0.86 – 1.14)	.87

eTable12 Footnote:
Analyses of associations between use of Acetylsalicylic acid (ASA), Metformin and Statin at time of RP and treatment for recurrence.

eTable12 Footnote continued:

Abbreviations: ASA, acetylsalicylic acid; nsBB, non-selective β-blocker; sBB, selective β-blocker; nonBB, no β-blocker
[a]Multivariate model adjusted for clinical tumor stage, pathological or clinical nodal stage, ISUP grade group, PSA interval and basic patients characteristics (age at RP, time from diagnosis to RP, year of RP and ECOG), follow up from 6months (183d after RP), and co-medication as indicated by table sub-headings.
[b]Use of nsBB, sBB, ASA, metformin, statin is included as covariates.
[c]Pooled results from 50 imputations; 11117, 1622 events
*this value is rounded up from .999
[d]Complete case analysis, 7147 cases, 979 events
[e]The only co-medication variable included is ASA
[f]The only co-medication variable included is metformin
[g]The only co-medication variable included is statin

eFigure1. Flow-chart illustrating the definition of drug users

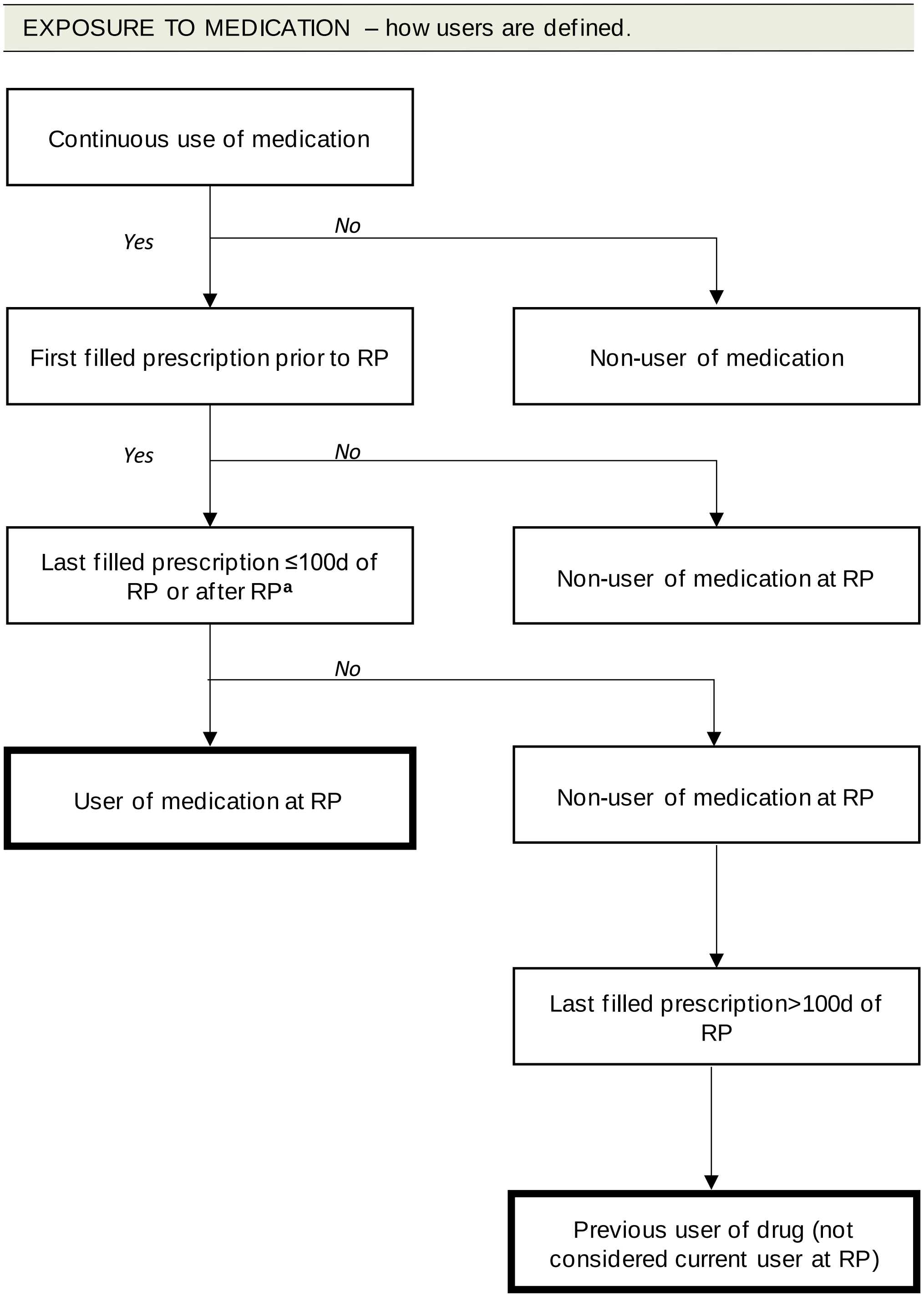

eFigure 1 Legend:

Flowchart of how current and previous use of a drug Is defined.
Abbreviations: RP, Radical prostatectomy
[a]100 days were chosen because regularly prescribed medications are usually given in quantities that cover 3 months.
